# Synthesis and Larvicidal Activity of Novel Thenoylhydrazide Derivatives

**DOI:** 10.1038/srep22977

**Published:** 2016-03-10

**Authors:** Gao-Peng Song, De-Kun Hu, Hao Tian, Ya-Sheng Li, Yun-Shen Cao, Hong-Wei Jin, Zi-Ning Cui

**Affiliations:** 1 Guangdong Province Key Laboratory of Microbial Signals and Disease Control, South China Agricultural University, Guangzhou, 510642, China; 2 College of Materials and Energy, South China Agricultural University, Guangzhou 510642, China; 3 State Key Laboratory of Natural and Biomimetic Drugs, School of Pharmaceutical Sciences, Peking University, Beijing 100191, China

## Abstract

A pair of chemical isomeric structures of novel *N*-*tert*-butylphenyl thenoylhydrazide compounds **I** and **II** were designed and synthesized. Their structures were characterized by MS, IR, ^1^H NMR, elemental analysis and X-ray single crystal diffraction. The regioselectivity of the Meerwein arylation reaction and the electrophilic substitution reaction of *N*-*tert*-butyl hydrazine were studied by density functional theory (DFT) quantum chemical method. The larvicidal tests revealed that some compounds **I** had excellent larvicidal activity against *Culex pipiens pallens*. As the candidates of insect growth regulators (IGRs), the larval growth inhibition and regulation against *Culex pipiens pallens* were examined for some compounds, especially **I1** and **I7**. Compounds **I1** and **I7** were further indicated as an ecdysteroid agonist by reporter gene assay on the *Spodoptera frugiperda* cell line (Sf9 cells). Finally, a molecular docking study of compound **I7** was conducted, which was not only beneficial to understand the structure-activity relationship, but also useful for development of new IGRs for the control of mosquitos.

Diacylhydrazines were as molting hormone analogs by mimicing the mode of 20-hydroxyecdysone (20E)^1^,^2^ since *N’*-*tert-*butyl-*N*,*N’*- dibenzoylhydrazine (RH-5849) was reported as the first nonsteroidal ecdysone agonist in the mid of 1980s^3^,^4^ (Fig. 1). The subsequent RH-5992 (tebufenozide)^5^,^6^, RH-0345 (halofenozide)^7^,^8^ and RH-2485 (methoxyfenozide)^9^,^10^ were developed. As a new class of insect growth regulators (IGRs), diacylhydrazines have attracted considerable attention in recent years^11^,^12^,^13^,^14^,^15^,^16^,^17^,^18^,^19^,^20^,^21^,^22^,^23^,^24^,^25^. The Fujita and Nakagawa group^11^,^12^,^13^,^14^,^15^,^16^ was focused on the modification of substituents on the di-benzoyl groups, and QSAR (Quantitative Structure Activity Relationship) research was studied based on the molecular libraries to guide the further design of the molting hormone analogs. The low water and organic solvent solubility limited the biological efficacy of the diacylhydraines. Therefore, some studies were focused on reconstruction and modification of the *N’*-*tert-*butyl linker to improve this drawback^17^,^18^,^19^,^20^,^21^,^22^,^23^,^24^,^25^. The replacement of the benzene ring with benzoheterocycles containing oxygen^26^,^27^,^28^,^29^,^30^ brings the recent products ANS-118 (chromafenozide)^31^,^32^, and JS-118^33^,^34^.

The complex crystal structures of the ligand-binding domains (LBDs) of ecdysone receptor (EcR) from *Heliothis virescens* (HvEcR) with ponasterone A (one of the ecdysteroids) and BYI06830 (a nonsteroidal agonist) were reported^35^. As demonstrated in the crystal structures, the binding pockets of HvEcR for BYI06830 and ponasterone A were partially overlapped. However, the aromatic ring of BYI06830 away from the *t*-butyl group was oriented in an extra pocket that was not occupied by ponasterone A. The difference between the binding modes revealed new insights to improve the agonist activity of diacylhydrazines by modifying the aromatic ring moiety away from the *t*-butyl group. Structure-activity relationship studies elucidated that hydrogen bonds between the amide N-H group of diacylhydrazines and amino acid residues of the LBD of EcR played a critical role in bioactivity^35^,^36^. As indicated in 3D QSAR studies^37^,^38^,^39^, physical chemical properties, such as electrostatic, steric and hydrophobic parameters of the ligands, were key factors affecting the binding affinity of molting hormone agonists.

Encouraged by the above discovery, in our previous work^40^,^41^,^42^,^43^,^44^,^45^,^46^, a series of diacylhydrazines with broad spectrum biological activity were designed and synthesized, and interestingly, the intermediate *N*-*tert*-butyl mono-acylhydrazide showed much better insecticidal activity than that of the diacylhydrazide^44^.

Thiophene is the bioisoster of furan, which also possesses diverse and significant bioactivities^47^,^48^,^49^,^50^. For the purpose of discovering novel lead compounds with bioactivity, a pair of chemical isomeric structures of *N*-*tert*-butylphenyl thenoylhydrazide compounds **I** and **II** were designed and synthesized in this letter. Their larvicidal activity was evaluated.

## Materials and Methods

### Instruments

All the melting points were determined with a Cole-Parmer melting point apparatus (Cole-Parmer, Vernon Hills, Illinois, USA) while the thermometer was uncorrected. IR spectra were recorded on a Nicolet NEXUS-470 FTIR spectrometer (International Equipment Trading Ltd., Vernon Hills, Illinois, USA) with KBr pellets. ^1^H NMR spectra were recorded with a Bruker DPX300 instrument (Bruker, Fallanden, Switzerland), while tetramethylsilane was used as an internal standard. Analytical thin-layer chromatography (TLC) was carried out on silica gel 60 F254 plates, and spots were visualized with ultraviolet (UV) light. Elemental analyses (C, H and N) were carried out with a Flash EA 1112 elemantal analyzer (Thermo Finnigan, Bremen, Germany). Mass spectra were measured on a Bruker ESQUIRE-LC spectrometer (Bruker, Fallanden, Switzerland). The X-ray crystal diffraction data were collected with a Rigaku Saturn diffractometer (Japan) at 294(2) K and the crystal structures were calculated using the SHELXL program package and refined by full-matrix least squares procedures at Nankai University.

### Chemistry

#### Synthetic Procedures

General synthetic procedure for 3-(substituted phenyl)-2-thenoic acid. Preparation of different *3-(substituted phenyl)-2-thenoic acid* was performed as previously described^40^,^41^,^42^,^43^,^44^,^45^,^46^.

General synthetic procedure for N’-tert-butyl-N-[3-(substituted phenyl)-2-thenoyl] hydrazide (**I** and **II**). A mixture of 3-substituted phenyl-2-thenoic acid (0.05 mol) and thionyl chloride (0.15 mol) was refluxed in anhydrous benzene for 3 h. The excess of thionyl chloride and the solvent were distilled off, and the residues were dissolved in anhydrous dichloromethane.

A solution of dilute aqueous sodium hydroxide (10%, 16 mmol) was added dropwise to the stirred solution of *tert-*butylhydrazine hydrochloride (16 mmol) in dichloromethane (10 mL) at 0 °C. The reaction mixture was stirred for 30 min, followed by adding dropwise the solution of 3-(substituted phenyl)-2-thenoyl chloride (4 mmol) in dichloromethane (10 mL) and dilute aqueous sodium hydroxide (10%, 4 mmol) at 0 °C simultaneously. Then the mixture was stirred at room temperature for 5 h and filtered to obtain a yellow solution. The organic filtrate was washed with water and dried with anhydrous magnesium sulphate overnight. After removing the solvent, the residues were purified by flash column chromatography (40 × 250 mm) on silica gel using a mixture of petroleum ether (60–90 °C) and ethyl acetate as the eluent (*V*_petroleum ether_/*V*_ethyl acetate_ = 3/1) to obtain **I** and **II**.

### ***N*****’-(*****tert***-butyl)-3-(2-chlorophenyl)thiophene-2-carbohydrazide (I1)

White solid, yield, 58.0%; m.p. 187–188 °C; IR (KBr) *ν*_*max*_: 3431.7, 3212.2, 2985.0, 1644.8, 1614.8, 1583.2, 1540.1, 1489.7, 1431.5, 1416.9, 1354.5, 1204.6 cm^−1^. ^1^H NMR (300 MHz, CDCl_3_) 0.95 (s, 9H, *t*-Bu), 4.80 (s, 1H, NH-*t*-Bu), 6.78 (s, 1H, NHCO), 7.00–7.02 (m, 1H, ThioH), 7.48–7.55 (m, 3H, ThioH + 2PhH), 7.65–7.68 (m, 1H, PhH), 7.96–8.00 (m, 1H, PhH). ESIMS (*m/z*): 309.4 [M + H]^+^. Anal. Calcd. (%) for C_15_H_17_ClN_2_OS: C, 58.34; H, 5.55; N, 9.07. Found: C, 58.65; H, 5.74; N, 9.09.

### *N*-(*tert*-butyl)-3-(2-chlorophenyl)thiophene-2-carbohydrazide (II1)

White solid, yield, 25.8%; m.p. 201–202 °C; IR (KBr) *ν*_*max*_: 3446.2, 3237.1, 2981.4, 1675.9, 1601.7, 1576.1, 1536.3, 1453.2, 1346.1, 1254.4, 1221.7 cm^−1^. ^1^H NMR (300 MHz, CDCl_3_) 1.54 (s, 9H, *t*-Bu), 3.93 (s, 2H, NH_2_), 7.39–7.41 (m, 1H, ThioH), 7.51–7.57 (m, 2H, PhH), 7.66–7.69 (m, 1H, PhH), 7.96–8.01 (m, 2H, ThioH + PhH). ESIMS (*m/z*): 331.3 [M + Na]^+^. Anal. Calcd. (%) for C_15_H_17_ClN_2_OS: C, 58.34; H, 5.55; N, 9.07. Found: C, 58.22; H, 5.68; N, 8.87.

### ***N*** **’-(** ***tert***-butyl)-3-(3-chlorophenyl)thiophene-2-carbohydrazide (I2)

White solid, yield, 66.0%; m.p. 167–168 °C; IR (KBr) *ν*_*max*_: 3444.6, 3337.8, 3212.4, 2975.8, 1609.2, 1612.3, 1543.1, 1487.4, 1458.2, 1421.1, 1217.6 cm^−1^. ^1^H NMR (300 MHz, CDCl_3_) 0.96 (s, 9H, *t*-Bu), 4.80 (s, 1H, NH-*t*-Bu), 6.79 (s, 1H, NHCO), 6.99–7.02 (m, 1H, ThioH), 7.49–7.54 (m, 3H, ThioH + 2PhH), 7.86–7.89 (m, 1H, PhH), 7.94–7.96 (m,1H, PhH). ESIMS (*m/z*): 331.1 [M + Na]^+^. Anal. Calcd. (%) for C_15_H_17_ClN_2_OS: C, 58.34; H, 5.55; N, 9.07. Found: C, 58.19; H, 5.82; N, 9.13.

### *N*-(*tert*-butyl)-3-(3-chlorophenyl)thiophene-2-carbohydrazide (II2)

White solid, yield, 22.0%; m.p. 189–190 °C; IR (KBr) *ν*_*max*_: 3438.1, 3215.3, 2976.2, 1685.1, 1600.5, 1568.2, 1487.4, 1454.2, 1234.3 1201.2 cm^−1^. ^1^H NMR (300 MHz, CDCl_3_) 1.56 (s, 9H, *t*-Bu), 3.94 (s, 2H, NH_2_), 7.38–7.40 (m, 1H, ThioH), 7.51–7.55 (m, 2H, PhH), 7.86–7.89 (m, 1H, PhH), 7.97–8.02 (m,2H, ThioH + PhH). ESIMS (*m/z*): 331.3 [M + Na]^+^. Anal. Calcd. (%) for C_15_H_17_ClN_2_OS: C, 58.34; H, 5.55; N, 9.07. Found: C, 58.57; H, 5.38; N, 9.02.

### ***N*** **’-(** ***tert***-butyl)-3-(4-chlorophenyl)thiophene-2-carbohydrazide (I3)

White solid, yield, 64.1%; m.p. 187–188 °C; IR (KBr) *ν*_*max*_: 3440.2, 2994.1, 1623.5, 1573.2, 1531.4, 1473.7, 1421.0, 1216.4 cm^−1^. ^1^H NMR (300 MHz, DMSO-*d*_*6*_) 1.07 (s, 9H, *t*-Bu), 5.02 (s, 1H, NH-*t*-Bu), 7.47–7.53 (m, 2H, PhH), 7.57 (d, *J* = 5.04 Hz, 1H, ThioH), 7.70–7.76 (m, 2H, PhH), 7.89 (d, *J* = 5.04 Hz, 1H, ThioH), 9.91 (s, 1H, NHCO). ESIMS (*m/z*): 309.1 [M + H]^+^. Anal. Calcd. (%) for C_15_H_17_ClN_2_OS: C, 58.34; H, 5.55; N, 9.07. Found: C, 58.71; H, 5.79; N, 8.89.

### ***N*** **-(** ***tert***-butyl)-3-(4-chlorophenyl)thiophene-2-carbohydrazide (II3)

White solid, yield, 18.0%; m.p. 222–223 °C; IR (KBr) *ν*_*max*_: 3444.8, 3203.2, 2934.1, 1621.8, 1543.2, 1494.7, 1421.5, 1344.1, 1216.3 cm^−1^. ^1^H NMR (300 MHz, CDCl_3_) 1.54 (s, 9H, *t*-Bu), 3.93 (s, 2H, NH_2_), 7.22 (d, *J* = 5.02 Hz, 1H, ThioH), 7.33–7.37 (m, 2H, PhH), 7.53–7.58 (m, 2H, PhH), 7.97 (d, *J* = 5.04 Hz, 1H, ThioH). ESIMS (*m/z*): 331.4 [M + Na]^+^. Anal. Calcd. (%) for C_15_H_17_ClN_2_OS: C, 58.34; H, 5.55; N, 9.07. Found: C, 58.52; H, 5.34; N, 9.26.

### ***N*** **’-(** ***tert***-butyl)-3-(2-fluorophenyl)thiophene-2-carbohydrazide (I4)

White solid, yield, 55.8%; m.p. 109–110 °C; IR (KBr) *ν*_*max*_: 3445.5, 3245.9, 2971.1, 1653.9, 1539.3, 1486.9, 1444.3, 1307.5, 1212.6 cm^−1^. ^1^H NMR (300 MHz, CDCl_3_) 0.95 (s, 9H, *t*-Bu), 4.80 (s, 1H, NH-*t*-Bu), 6.78 (s, 1H, NHCO), 7.02 (d, *J* = 5.02 Hz, 1H, ThioH), 7.18–7.28 (m, 2H, PhH), 7.37–7.46 (m, 2H, PhH), 7.52 (d, *J* = 5.04 Hz, 1H, ThioH). ESIMS (*m/z*): 315.2 [M + Na]^+^. Anal. Calcd. (%) for C_15_H_17_FN_2_OS: C, 61.62; H, 5.86; N, 9.58. Found: C, 61.37; H, 5.78; N, 9.55.

### *N*-(*tert*-butyl)-3-(2-fluorophenyl)thiophene-2-carbohydrazide (II4)

White solid, yield, 29.4%; m.p. 139–140 °C; IR (KBr) *ν*_*max*_: 3443.7, 3322.8, 3213.3, 2927.5, 1583.5, 1453.1, 1397.6, 1203.8 cm^−1^. ^1^H NMR (300 MHz, CDCl_3_) 1.54 (s, 9H, *t*-Bu), 3.95 (s, 2H, NH_2_), 7.14–7.19 (m, 2H, PhH), 7.39–7.43 (m, 2H, ThioH + PhH), 7.63–7.69 (m, 1H, PhH), 8.00–8.02 (m, 1H, ThioH). ESIMS (*m/z*): 315.1 [M + Na]^+^. Anal. Calcd. (%) for C_15_H_17_FN_2_OS: C, 61.62; H, 5.86; N, 9.58. Found: C, 61.65; H, 5.63; N, 9.44.

### ***N*** **’-(** ***tert***-butyl)-3-(3-fluorophenyl)thiophene-2-carbohydrazide (I5)

White solid, yield, 58.6%; m.p. 120–121 °C; IR (KBr) *ν*_*max*_: 3424.1, 3246.2, 2971.0, 1654.8, 1614.8, 1583.1, 1540.1, 1479.7, 1441.5, 1308.2, 1189.1 cm^−1^. ^1^H NMR (300 MHz, CDCl_3_) 0.96 (s, 9H, *t*-Bu), 4.80 (s, 1H, NH-*t*-Bu), 6.77 (s, 1H, NHCO), 7.01 (d, *J* = 5.02 Hz, 1H, ThioH), 7.13–7.19 (m, 2H, PhH), 7.21–7.25 (m, 1H, PhH),7.42–7.51 (m, 2H, ThioH + PhH). ESIMS (*m/z*): 293.2 [M + H]^+^. Anal. Calcd. (%) for C_15_H_17_FN_2_OS: C, 61.62; H, 5.86; N, 9.58. Found: C, 61.98; H, 5.86; N, 9.62.

### *N*-(*tert*-butyl)-3-(3-fluorophenyl)thiophene-2-carbohydrazide (II5)

White solid, yield, 24.5%; m.p. 143–144 °C; IR (KBr) *ν*_*max*_: 3416.6, 3244.2, 2973.8, 1650.4, 1539.9, 1498.7, 1478.7, 1444.5, 1306.3, 1215.8 cm^−1^. ^1^H NMR (300 MHz, CDCl_3_) 1.54 (s, 9H, *t*-Bu), 3.96 (s, 2H, NH_2_), 7.12–7.19 (m, 2H, PhH), 7.22–7.25 (m, 1H, PhH), 7.39–7.41 (m, 1H, ThioH), 7.47–7.51 (m, 1H, PhH), 7.96–7.98 (m, 1H, ThioH). ESIMS (*m/z*): 315.2 [M + Na]^+^. Anal. Calcd. (%) for C_15_H_17_FN_2_OS: C, 61.62; H, 5.86; N, 9.58. Found: C, 61.77; H, 5.99; N, 9.71.

### ***N*** **’-(** ***tert***-butyl)-3-(4-fluorophenyl)thiophene-2-carbohydrazide (I6)

White solid, yield, 60.3%; m.p. 138–139 °C; IR (KBr) *ν*_*max*_: 3431.9, 3242.9, 2971.0, 1655.7, 1542.1, 1496.4, 1480.5, 1307.6, 1264.4 cm^−1^. ^1^H NMR (300 MHz, CDCl_3_) 0.96 (s, 9H, *t*-Bu), 4.79 (s, 1H, NH-*t*-Bu), 6.75 (s, 1H, NHCO), 7.00 (d, *J* = 5.02 Hz, 1H, ThioH), 7.14–7.21 (m, 2H, PhH), 7.39–7.45 (m, 2H, PhH), 7.49 (d, *J* = 5.02 Hz, 1H, ThioH). ESIMS (*m/z*): 315.3 [M + Na]^+^. Anal. Calcd. (%) for C_15_H_17_FN_2_OS: C, 61.62; H, 5.86; N, 9.58. Found: C, 61.93; H, 5.95; N, 9.67.

### *N*-(*tert*-butyl)-3-(4-fluorophenyl)thiophene-2-carbohydrazide (II6)

White solid, yield, 22.5%; m.p. 109–110 °C; IR (KBr) *ν*_*max*_: 3354.5, 2964.1, 1618.5, 1536.1, 1498.4, 1448.0, 1391.0, 1269.0, 1203.5 cm^−1^. ^1^H NMR (300 MHz, CDCl_3_) 1.53 (s, 9H, *t*-Bu), 3.94 (s, 2H, NH_2_), 7.04–7.10 (m, 2H, PhH), 7.17 (d, *J* = 5.02 Hz, 1H, ThioH), 7.56–7.61 (m, 2H, PhH), 7.96 (d, *J* = 5.02 Hz, 1H, ThioH). ESIMS (*m/z*): 293.2 [M + H]^+^. Anal. Calcd. (%) for C_15_H_17_FN_2_OS: C, 61.62; H, 5.86; N, 9.58. Found: C, 61.68; H, 5.89; N, 9.62.

### ***N*** **’-(** ***tert***-butyl)-3-(2,4-difluorophenyl)thiophene-2-carbohydrazide (I7)

White solid, yield, 60.5%; m.p. 148–149 °C; IR (KBr) *ν*_*max*_: 3414.7, 3274.6, 2974.8, 1651.5, 1544.6, 1463.5, 1446.0, 1313.6, 1238.8 cm^−1^. ^1^H NMR (300 MHz, DMSO-*d*_*6*_) 0.93 (s, 9H, *t*-Bu), 4.74 (s, 1H, NH-*t*-Bu), 7.11–7.17 (m, 2H, ThioH + PhH), 7.25–7.32 (m, 1H, PhH), 7.43–7.51 (m, 1H, PhH), 7.77 (d, *J* = 5.04 Hz, 1H, ThioH), 9.38 (s, 1H, NHCO). ESIMS (*m/z*): 311.4 [M + H]^+^. Anal. Calcd. (%) for C_15_H_16_F_2_N_2_OS: C, 58.05; H, 5.20; N, 9.03. Found: C, 57.88; H, 5.27; N, 9.31.

### *N*-(*tert*-butyl)-3-(2,4-difluorophenyl)thiophene-2-carbohydrazide (II7)

White solid, yield, 28.8%; m.p. 194–195 °C; IR (KBr) *ν*_*max*_: 3357.9, 2970.2, 1616.3, 1572.7, 1465.2, 1435.2, 1392.3, 1365.5, 1253.9, 1203.7 cm^−1^. ^1^H NMR (300 MHz, DMSO-*d*_*6*_) 1.45 (s, 9H, *t*-Bu), 5.13 (s, 2H, NH_2_), 7.19–7.25 (m, 1H, PhH), 7.36–7.45 (m, 2H, ThioH + PhH), 7.79–7.85 (m, 2H, ThioH + PhH). ESIMS (*m/z*): 333.3 [M + Na]^+^. Anal. Calcd. (%) for C_15_H_16_F_2_N_2_OS: C, 58.05; H, 5.20; N, 9.03. Found: C, 58.20; H, 5.24; N, 9.12.

### ***N*** **’-(** ***tert***-butyl)-3-(2,6-difluorophenyl)thiophene-2-carbohydrazide (I8)

White solid, yield, 61.8%; m.p. 180–181 °C; IR (KBr) *ν*_*max*_: 3441.1, 3223.2, 2956.2, 1623.4, 1600.3, 1584.2, 1552.3, 1475.7, 1421.5, 1206.7 cm^−1^. ^1^H NMR (300 MHz, DMSO-*d*_*6*_) 1.07 (s, 9H, *t*-Bu), 5.07 (s, 1H, NH-*t*-Bu), 7.24–7.33 (m, 2H, ThioH + PhH), 7.44–7.55 (m, 2H, ThioH + PhH), 7.88 (d, *J* = 5.04 Hz, 1H, ThioH), 9.99 (s, 1H, NHCO). ESIMS (*m/z*): 333.1 [M + Na]^+^. Anal. Calcd. (%) for C_15_H_16_F_2_N_2_OS: C, 58.05; H, 5.20; N, 9.03. Found: C, 57.97; H, 5.16; N, 9.10.

### *N*-(*tert*-butyl)-3-(2,6-difluorophenyl)thiophene-2-carbohydrazide (II8)

White solid, yield, 29.5%; m.p. 165–166 °C; IR (KBr) *ν*_*max*_: 3231.2, 2943.0, 1652.3, 1604.8, 1564.1, 1520.1, 1479.2, 1421.3, 1214.3 cm^−1^. ^1^H NMR (300 MHz, CDCl_3_) 1.54 (s, 9H, *t*-Bu), 3.96 (s, 2H, NH_2_), 6.96–7.03 (m, 2H, PhH), 7.21–7.26 (m, 1H, PhH), 7.45–7.47 (m, 1H, ThioH), 8.02 (d, *J* = 5.04 Hz, 1H, ThioH). ESIMS (*m/z*): 333.3 [M + Na]^+^. Anal. Calcd. (%) for C_15_H_16_F_2_N_2_OS: C, 58.05; H, 5.20; N, 9.03. Found: C, 58.23; H, 5.03; N, 9.06.

### ***N*** **’-(** ***tert***-butyl)-3-(2-nitrophenyl)thiophene-2-carbohydrazide (I9)

Yellow solid, yield, 61.8%; m.p. 180–181 °C; IR (KBr) *ν*_*max*_: 3451.2, 3286.1, 2987.2, 1665.8, 1624.8, 1593.2, 1552.1, 1457.7, 1426.5, 1406.9, 1344.5, 1205.7 cm^−1^. ^1^H NMR (300 MHz, CDCl_3_) 0.97 (s, 9H, *t*-Bu), 4.79 (s, 1H, NH-*t*-Bu), 6.78 (s, 1H, NHCO), 7.01–7.03 (m, 1H, ThioH), 7.68–7.73 (m, 1H, PhH), 7.51–7.53 (m, 1H, ThioH), 7.79–7.83 (m, 1H, PhH), 8.00–8.05 (m, 2H, PhH). ESIMS (*m/z*): 320.2 [M + H]^+^. Anal. Calcd. (%) for C_15_H_17_N_3_O_3_S: C, 56.41; H, 5.37; N, 13.16. Found: C, 56.22; H, 5.43; N, 13.29.

### *N*-(*tert*-butyl)-3-(2-nitrophenyl)thiophene-2-carbohydrazide (II9)

Yellow solid, yield, 22.3%; m.p. 174–175 °C; IR (KBr) *ν*_*max*_: 3453.7, 3227.1, 2991.3, 1653.2, 1617.7, 1585.2, 1537.4, 1484.4, 1380.5, 1207.6 cm^−1^. ^1^H NMR (300 MHz, CDCl_3_) 1.54 (s, 9H, *t*-Bu), 3.96 (s, 2H, NH_2_), 7.41–7.43 (m, 1H, ThioH), 7.67–7.71 (m, 1H, PhH), 7.78–7.82 (m, H, PhH), 8.00–8.06 (m, 3H, ThioH + 2PhH). ESIMS (*m/z*): 342.1 [M + Na]^+^. Anal. Calcd. (%) for C_15_H_17_N_3_O_3_S: C, 56.41; H, 5.37; N, 13.16. Found: C, 56.09; H, 5.47; N, 13.31.

### ***N*** **’-(** ***tert***-butyl)-3-(3-nitrophenyl)thiophene-2-carbohydrazide (I10)

Yellow solid, yield, 57.3%; m.p. 139–140 °C; IR (KBr) *ν*_*max*_: 3451.5, 3222.1, 2972.0, 1634.7, 1627.2, 1545.5, 1538.0, 1462.3, 1421.4, 1354.5 cm^−1^. ^1^H NMR (300 MHz, CDCl_3_) 0.96 (s, 9H, *t*-Bu), 4.78 (s, 1H, NH-*t*-Bu), 6.75 (s, 1H, NHCO), 7.01–7.03 (m, 1H, ThioH), 7.50–7.52 (m, 1H, ThioH), 7.80–7.84 (m, 1H, PhH), 8.26–8.31(m, 2H, PhH), 8.68–8.71 (m, 1H, PhH). ESIMS (*m/z*): 342.3 [M + Na]^+^. Anal. Calcd. (%) for C_15_H_17_N_3_O_3_S: C, 56.41; H, 5.37; N, 13.16. Found: C, 56.51; H, 5.27; N, 13.04.

### *N*-(*tert*-butyl)-3-(3-nitrophenyl)thiophene-2-carbohydrazide (II10)

Yellow solid, yield, 20.4%; m.p. 160–161 °C; IR (KBr) *ν*_*max*_: 3451.4, 3421.3, 2995.7, 1614.4, 1593.7, 1546.1, 1521.2, 1452.6, 1411.5, 1371.6, 1214.3 cm^−1^. ^1^H NMR (300 MHz, CDCl_3_) 1.55 (s, 9H, *t*-Bu), 3.99 (s, 2H, NH_2_), 7.40–7.42 (m, 1H, ThioH), 7.79–7.83 (m, 2H, ThioH + PhH), 8.01–8.03 (m, 1H, ThioH), 8.17–8.26 (m, 2H, PhH), 8.63–8.66 (m, 1H, PhH). ESIMS (*m/z*): 342.1 [M + Na]^+^. Anal. Calcd. (%) for C_15_H_17_N_3_O_3_S: C, 56.41; H, 5.37; N, 13.16. Found: C, 56.39; H, 5.51; N, 13.23.

### ***N*** **’-(** ***tert***-butyl)-3-(4-nitrophenyl)thiophene-2-carbohydrazide (I11)

Yellow solid, yield, 64.1%; m.p. 181–182 °C; IR (KBr) *ν*_*max*_: 3451.4, 3222.5, 2995.0, 1654.9, 1624.2, 1593.4, 1584.2, 1540.1, 1500.9, 1434.7, 1350.0, 1216.3 cm^−1^. ^1^H NMR (300 MHz, DMSO-*d*_*6*_) 1.05 (s, 9H, *t*-Bu), 5.10 (s, 1H, NH-*t*-Bu), 7.71 (d, *J* = 5.04 Hz, 1H, ThioH), 7.82 (d, *J* = 5.04 Hz, 1H, ThioH), 7.96–8.01(m, 2H, PhH), 8.26–8.30 (m, 2H, PhH), 10.00 (s, 1H, NHCO). ESIMS (*m/z*): 342.3 [M + Na]^+^. Anal. Calcd. (%) for C_15_H_17_N_3_O_3_S: C, 56.41; H, 5.37; N, 13.16. Found: C, 56.64; H, 5.57; N, 13.01.

### *N*-(*tert*-butyl)-3-(4-nitrophenyl)thiophene-2-carbohydrazide (II11)

Yellow solid, yield, 19.6%; m.p. 166–167 °C; IR (KBr) *ν*_*max*_: 3449.4, 3216.2, 2975.5, 1642.6, 1593.4, 1543.5, 1434.2, 1415.5, 1344.5, 1214.6 cm^−1^. ^1^H NMR (300 MHz, CDCl_3_) 1.56 (s, 9H, *t*-Bu), 3.98 (s, 2H, NH_2_), 7.40 (d, *J* = 5.04 Hz, 1H, ThioH), 7.75–7.79 (m, 2H, PhH), 8.02 (d, *J* = 5.02 Hz, 1H, ThioH), 8.23–8.26 (m, 2H, PhH). ESIMS (*m/z*): 320.1 [M + H]^+^. Anal. Calcd. (%) for C_15_H_17_N_3_O_3_S: C, 56.41; H, 5.37; N, 13.16. Found: C, 56.47; H, 5.19; N, 13.25.

### ***N*** **’-(** ***tert***-butyl)-3-phenylthiophene-2-carbohydrazide (I12)

White solid, yield, 56.8%; m.p. 154–155 °C; IR (KBr) *ν*_*max*_: 3432.4, 2965.7, 1614.3, 1553.0, 1510.2, 1479.6, 1421.1, 1205.4 cm^−1^. ^1^H NMR (300 MHz, CDCl_3_) 0.97 (s, 9H, *t*-Bu), 4.76 (s, 1H, NH-*t*-Bu), 6.77 (s, 1H, NHCO), 6.99–7.01 (m, 1H, ThioH), 7.45–7.58 (m, 4H, ThioH + 3PhH), 7.90–7.94 (m, 2H, PhH). ESIMS (*m/z*): 297.1 [M + Na]^+^. Anal. Calcd. (%) for C_15_H_18_N_2_OS: C, 65.66; H, 6.61; N, 10.21. Found: C, 65.89; H, 6.73; N, 10.01.

### *N*-(*tert*-butyl)-3-phenylthiophene-2-carbohydrazide (II12)

White solid, yield, 15.3%; m.p. 160–161 °C; IR (KBr) *ν*_*max*_: 3441.2, 3213.5, 2974.0, 1614.7, 1563.4, 1510.1, 1476.5, 1432.2, 1406.9, 1351.4, 1211.3 cm^−1^. ^1^H NMR (300 MHz, CDCl_3_) 1.57 (s, 9H, *t*-Bu), 3.96 (s, 2H, NH_2_), 7.41–7.50 (m, 4H, ThioH + 3PhH), 7.94–8.02 (m, 3H, ThioH + 2PhH). ESIMS (*m/z*): 297.3 [M + Na]^+^. Anal. Calcd. (%) for C_15_H_18_N_2_OS: C, 65.66; H, 6.61; N, 10.21. Found: C, 65.78; H, 6.54; N, 10.42.

### ***N*** **’-(** ***tert***-butyl)-3-(*p*-tolyl)thiophene-2-carbohydrazide (I13)

White solid, yield, 58.6%; m.p. 149–150 °C; IR (KBr) *ν*_*max*_: 3438.7, 3202.2, 2995.4, 1654.9, 1604.3, 1573.1, 1520.1, 1445.9, 1352.5, 1206.1 cm^−1^. ^1^H NMR (300 MHz, CDCl_3_) 0.97 (s, 9H, *t*-Bu), 2.37 (s, 3H, CH_3_), 4.79 (s, 1H, NH-*t*-Bu), 6.78 (s, 1H, NHCO), 6.99–7.01 (m, 1H, ThioH), 7.26–7.30 (m, 2H, PhH), 7.49–7.51 (m, 1H, ThioH), 7.79–7.82 (m, 2H, PhH). ESIMS (*m/z*): 289.2 [M + H]^+^. Anal. Calcd. (%) for C_16_H_20_N_2_OS: C, 66.63; H, 6.99; N, 9.71. Found: C, 66.41; H, 7.14; N, 9.63.

### *N*-(*tert*-butyl)-3-(*p*-tolyl)thiophene-2-carbohydrazide (II13)

White solid, yield, 14.8%; m.p. 168–169 °C; IR (KBr) *ν*_*max*_: 3221.4, 2974.3, 1656.5, 1616.3, 1582.7, 1531.1, 1479.7, 1421.5, 1210.6 cm^−1^. ^1^H NMR (300 MHz, CDCl_3_) 1.54 (s, 9H, *t*-Bu), 2.37 (s, 3H, CH_3_), 3.96 (s, 2H, NH_2_), 7.19–7.21 (m, 1H, ThioH), 7.38–7.42 (m, 2H, PhH), 7.78–7.83 (m, 2H, PhH), 8.00–8.02 (m, 1H, ThioH). ESIMS (*m/z*): 311.1 [M + Na]^+^. Anal. Calcd. (%) for C_16_H_20_N_2_OS: C, 66.63; H, 6.99; N, 9.71. Found: C, 66.90; H, 7.26; N, 9.54.

### ***N*** **’-(** ***tert***-butyl)-3-(4-methoxyphenyl)thiophene-2-carbohydrazide (I14)

White solid, yield, 59.5%; m.p. 167–168 °C; IR (KBr) *ν*_*max*_: 3441.7, 3232.3, 2972.0, 1604.3, 1563.6, 1520.2, 1479.1, 1417.6, 1334.4 cm^−1^. ^1^H NMR (300 MHz, CDCl_3_) 0.97 (s, 9H, *t*-Bu), 3.84 (s, 3H, OCH_3_), 4.79 (s, 1H, NH-*t*-Bu), 6.78 (s, 1H, NHCO), 7.01–7.03 (m, 1H, ThioH), 7.08–7.13 (m, 2H, PhH), 7.48–7.50 (m, 1H, ThioH), 7.83–7.88 (m, 2H, PhH). ESIMS (*m/z*): 327.1 [M + Na]^+^. Anal. Calcd. (%) for C_16_H_20_N_2_O_2_S: C, 63.13; H, 6.62; N, 9.20. Found: C, 63.38; H, 6.83; N, 9.05.

### *N*-(*tert*-butyl)-3-(4-methoxyphenyl)thiophene-2-carbohydrazide (II14)

White solid, yield, 14.1%; m.p. 183–184 °C; IR (KBr) *ν*_*max*_: 3331.3, 3216.2, 2974.6, 1634.2, 1605.8, 1562.9, 1463.5, 1412.9, 1362.2, 1213.7 cm^−1^. ^1^H NMR (300 MHz, CDCl_3_) 1.54 (s, 9H, *t*-Bu), 3.84 (s, 3H, OCH_3_), 3.96 (s, 2H, NH_2_), 7.08–7.13 (m, 2H, PhH), 7.18–7.20 (m, 1H, ThioH), 7.83–7.88 (m, 2H, PhH), 7.99–8.02 (m, 1H, ThioH). ESIMS (*m/z*): 327.3 [M + Na]^+^. Anal. Calcd. (%) for C_16_H_20_N_2_O_2_S: C, 63.13; H, 6.62; N, 9.20. Found: C, 62.99; H, 6.74; N, 9.28.

### ***N*** **’-(** ***tert***-butyl)-3-(4-bromophenyl)thiophene-2-carbohydrazide (I15)

White solid, yield, 70.4%; m.p. 193–194 °C; IR (KBr) *ν*_*max*_: 3242.2, 2952.3, 1654.6, 1608.8, 1547.2, 1483.6, 1435.5, 1346.5, 1202.1 cm^−1^. ^1^H NMR (300 MHz, CDCl_3_) 0.97 (s, 9H, *t*-Bu), 4.79 (s, 1H, NH-*t*-Bu), 6.78 (s, 1H, NHCO), 6.99–7.02 (m, 1H, ThioH), 7.33–7.36 (m, 2H, 2PhH), 7.49–7.52 (m, 1H, ThioH), 7.66–7.69 (m, 2H, PhH). ESIMS (*m/z*): 353.1 [M + H]^+^. Anal. Calcd. (%) for C_15_H_17_BrN_2_OS: C, 51.00; H, 4.85; N, 7.93. Found: C, 51.28; H, 5.01; N, 7.74.

### *N*-(*tert*-butyl)-3-(4-bromophenyl)thiophene-2-carbohydrazide (II15)

White solid, yield, 19.4%; m.p. 214–215 °C; IR (KBr) *ν*_*max*_: 3445.1, 3221.7, 2973.6, 1631.6, 1586.2, 1547.3, 1424.4, 1415.2, 1323.5, 1215.6 cm^−1^. ^1^H NMR (300 MHz, CDCl_3_) 1.52 (s, 9H, *t*-Bu), 3.94 (s, 2H, NH_2_), 7.18–7.20 (m, 1H, ThioH), 7.31–7.35 (m, 2H, PhH), 7.62–7.66 (m, 2H, PhH), 7.99–8.01 (m, 1H, ThioH). ESIMS (*m/z*): 376.3 [M + Na]^+^. Anal. Calcd. (%) for C_15_H_17_BrN_2_OS: C, 51.00; H, 4.85; N, 7.93. Found: C, 50.81; H, 4.93; N, 8.08.

### Crystallography

Compound **I7** was recrystallized from methanol to give colorless crystals suitable for X-ray single crystal diffraction. Cell constants at 294(2) K were determined by a least-square fit to the setting parameters of independent reflections measured on a Bruker SMART^51^ 1000 CCD area-detector diffractometer with a graphite- monochromated Mo Kα radiation (*λ* = 0.071073 nm) and operating in the phi and scan modes. The structure was solved by the direct method with SHELXS-97^52^,^53^ and refined by the full-matrix least squares method on F2 data using SHELXL-97^53^,^54^. The empirical absorption corrections were applied to all intensity data. H atom of N-H was initially located in a different Fourier map and was refined with the restraint *U*iso(H) = 1.2 *U*eq(N). Other H atoms were positioned geometrically and refined using a riding model, with d(C…H) = 0.093–0.097 nm and *U*iso(H) = 1.2 *U*eq(C) or 1.5 *U*eq(C-methyl). The crystal data in CIF format have been deposited at the Cambridge Crystallographic Data Centre with deposition number CCDC 935115.

### Computational Method

Structures of 2-furoic acid, 2-thenoic acid, and *tert*-butyl hydrazine were calculated with density functional theory (DFT) quantum chemical method by using Gaussian 09 program package^55^. Equilibrium geometries of all the three molecules were fully optimized at the B3LYP/6-311G (d, p) level of theory^56^. Vibrational frequencies, calculated at the same level, were used to determine the nature of the stationary points. The charge densities of all the atoms in the three molecules were acquired.

### Bioassay

#### Larvicidal Activity

Assessments were made on a dead/alive basis. Evaluations are based on a percentage scale of 0–100, which 0 equals no activity and 100 equals total kill. The bioassay was repeated three times, and the result of bioactivity was the average of these replicates. The commercialized insecticide RH-5849 was tested as a control under the same condition. EXCEL2007 was applied to analyze bioassay data. The LC_50_ values of some active title compounds were evaluated using logit analysis and the results were analyzed using the statistical data processing system (DPS, 10.15, Zhejiang, China).

#### Larvicidal Activity against Mosquito (*Culex pipiens pallens*)

The larvicidal activity was evaluated at the preliminary test concentration of 10 μg mL^−1^ against the fourth-instar *Culex pipiens pallens* by the water immersion method^44^ under conditions of (27 ± 2) °C, photoperiod of 10:14 (light:dark), and relative humidity 50–70%. All the test beakers containing twenty *Culex pipiens pallens* were evaluated 8 days after treatment. The results were recorded by average percentage mortality.

#### Larvicidal Activity against Oriental Armyworm (*Mythimna separata*)

The larvicidal activity of the title compounds against oriental armyworm was evaluated by foliar application^19^,^21^. For the foliar armyworm tests, individual corn leaves were placed on moistened pieces of filter paper in Petri dishes. The leaves were then sprayed with the test solution at the preliminary test concentration of 200 μg mL^−1^ and allowed to dry. The dishes were infested with 10 fourth-instar oriental armyworm larvae. Percentage mortalities were evaluated 4 days after treatment.

#### Larvicidal Activity against Diamondback Moth (*Plutella xylostella*)

The larvicidal activity of the title compounds against diamondback moth was tested by leaf-dip method^23^,^25^. Leaf disks (1.8 cm diameter) were cut from fresh cabbage leaves and then were dipped into the test solution at the preliminary test concentration of 200 μg mL^−1^ for 15 s. After air-drying, the treated leaf disks were placed in a Petri dish (9 cm diameter) lined with a piece of filter paper, and then 10 second-instar diamondback moth larvae were transferred to the Petri dish. Percentage mortalities were evaluated 6 days after treatment.

#### Larval growth inhibition and regulation against *Culex pipiens pallens* determined by water immersion method

The title compounds were dissolved in acetone and diluted to the required concentration of 10 μg mL^−1^ as test solutions. Every 20 second-instar *Culex pipiens pallens* larvae were weighted in 50 mL beaker with exact quantity of water and the tested compounds. The water immersion method was applied referring to the previous procedure^44^. The weight of the treated larvae was recorded after a 72 h treatment and the larvae were reared until pupation and adult emergence. The duration of pupal and larval stages, and adult eclosion rate were recorded.

#### Agonistic activity determined by reporter gene assay

Sf9 cells (derived from ovary of *S. frugiperda* and obtained from South China Agricultural University, China) were used for the reporter gene assay. Cells were cultured in HyClone SFX-Insect serum-free insect culture medium (Thermo Fisher Scientific, China) supplying with 5% fetal calf serum at 27.5 °C. A reporter plasmid pBmbA/hsp27/gfp, referred as ERE-b.act-GFP, was composed of seven copies of the ERE derived from *D. melanogaster* hsp27 promoter, and a promoter region of actin A3 gene of *B. mori*, which was followed by an EGFP coding gene. This plasmid was constructed for detecting ecdysone agonists dependent activation of transcription by Dr. Swevers *et al*.^57^.

The plasmid was purified by Omega Endo-free Plasmid Midi Kit (Omega Bio-Tek, Inc., China) after amplified in *Escherichia coli* according to the manufacturer. Then the plasmids were merged with MegaTran 1.0 (OriGene, Beijing, China) as complex and gently added into the cells (5 × 10^4^ per well) in each well of 24-well plates. After incubation for 24 h, the test compounds (dissolved in DMSO) were diluted with culture medium and added into the cells for another 24 h incubation. The induced GFP fluorescence on the living cells was observed directly using Olympus CKX41 inverted microscope (Aizu, Japan). The micrographs were collected with ProgRes CF digital camera system (Jenoptik, Germany). Then, cells in each well were collected and were gently washed twice with 250 μL PBS and transferred to black 96-well plates at a density of 3 × 10^4^ cells/mL. Fluorescence intensity of the cells was recorded by a 1420 Multilabel Counter Victor 3V (Perkinelmer, Massachusetts, America), and corrected by auto-fluorescence and background fluorescence in the absence of compounds^58^.

#### Molecular modeling and docking study

**Figure 1:**
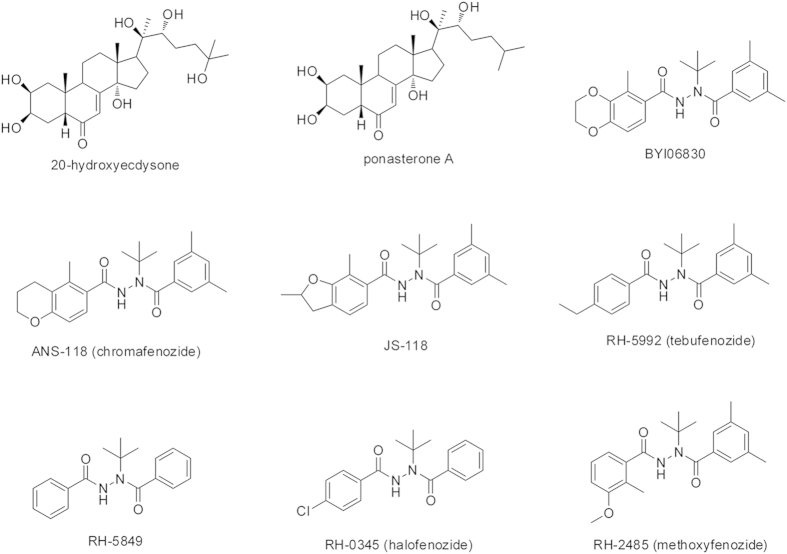
The structures of ecdysteroids and nonsteroidal ecdysone agonists.

The ligand binding domain (LBD) of *Culex pipiens* EcR was constructed on the Swiss Model website (http://swissmodel.expasy.org/)^59^,^60^,^61^. The primary sequence was derived from the NCBI database (NCBI accession number: XP_001844581). The sequence of *Culex pipiens* EcR was aligned with HvEcR with an identity of 76.65% (shown in Supplementary data Fig. 1S). The chain D of the crystal structure of the ligand binding domain of HvEcR (PDB: 1R20) was selected as the template for homology modeling building^62^,^63^. The constructed modeling was evaluated by QMEAN, with the value of 0.63, as a reasonable modeling^64^. The downloaded structure was qualified and employed for the docking study. Molecular docking was performed using Surflex-Dock protocol in Sybyl 8.0 with the MMFF94 force field to evaluate the potential molecular binding mode between the synthesized compound and LBD of *Culex pipiens* EcR.

The three dimensional structure of EcR LBD was constructed and refined with MMFF94 by energy minimization and defined as a receptor. The active site was defined based on the ligand binding location of BYI06830 (the original ligand) with a radius of 10 Å. Once the compound was docked into the active site, a simulated annealing method was conducted following the default parameters and programs. Finally, 20 molecular docking poses were saved and ranked according to their dock score function. The pose with the lowest interaction energy was considered as the best one. The interaction energy of the docked LBD of EcR with compound was −45.238 kcals mol^−1^ (pKd 6.54, crash −0.32, and polar 2.29). To obtain more insights into the ligand-receptor interaction, the binding modes of compound **I7** docked to the active site of EcR were exhibited and shown.

## Results and Discussion

### Chemistry

**Figure 2:**
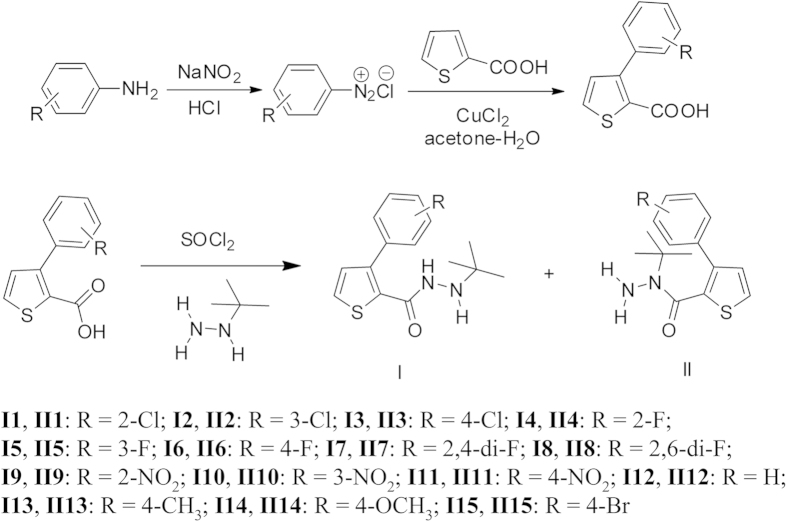
General Synthetic Procedure for Title compounds **I** and **II**.

By the method of Meerwein arylation using copper(II)-catalyzed decomposition of diazonium salts, a series of 5-substituted phenyl-2-furoic acid were prepared in good yields in our previous work. The nucleophilic reaction with 2-furoic acid often exhibited a high regioselectivity at the 5-position of furan ring. When we change the reagent from 2-furoic acid to 2-thenoic acid, the nucleophilic reaction still kept a high regioselectivity, but the reactive position changed to the 3-position of thiophene ring (Fig. 2). It could be confirmed by the X-ray single crystal diffraction of compound 2 and all the coupling constants of H-4 with H-5 (^*3*^*J*_*HH*_ = 5.04 Hz) in the ^1^H NMR data (see supplementary information for ^1^H NMR spectra).

**Figure 3:**
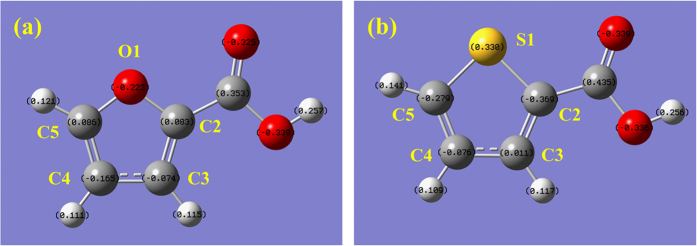
The charge density of all the atoms in 2-furoic acid (**a**) and 2-thenoic acid (**b**).

The DFT method was applied to calculate the optimized structures of 2-furoic and 2-thenoic acid. The charge density of all the atoms in both (Fig. 3) were obtained. The results showed that for 2-furoic acid, the charge density of C-5 (0.086) was positive, whereas that of C-3 (−0.074) and C-4 (−0.165) were negative, which meant that the nucleophilic reaction easily occurred at the C-5 position in 2-furoic acid. For 2-thenoic acid, the charge density of C-3 (0.011) was positive, while that of C-4 (−0.076) and C-5 (−0.279) were negative. It showed clearly that, compared to 2-furoic acid, the nucleophilic reaction selectively occurred at the C-3 position in 2-thenoic acid rather than C-5.

**Figure 4:**
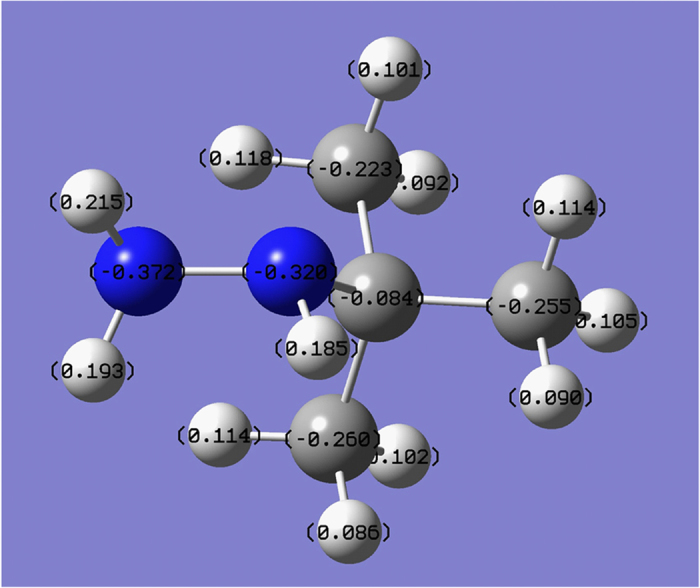
The charge density of all the atoms in *tert*-butyl hydrazine.

The same computational method was applied to calculate charge density of the two nitrogen atoms in the *tert*-butyl hydrazine (Fig. 4). The results showed that both of the nitrogen atoms were negative and the one connected with two hydrogen atoms had lower negative charge density (−0.372) than that of the one connected with the *tert*-butyl group (−0.320). Due to the bulky steric effect of the *tert*-butyl group and the low charge density, the electrophilic reaction was difficult to occur at the nitrogen atom connected to the *tert*-butyl group. That was the reason why all the title compounds **I** was the main product and had much better yield than compounds **II**.

The different effect factors, such as different substituted groups (Table S1), different bases (Table S2) and different molar ratio of reagents (Table S3), on the molar ratio of title compounds **I** and **II** were studied in this paper. It could be seen that the strong electron-withdrawing ability of the substituent led to the poor selectivity of the reaction. The sequence of the selectivity was 4-F < 4-NO_2_ < 4-Cl < H < 4-CH_3_ < 4-OCH_3_ (Table S1). And the position of the substituted group also affected the reactive selectivity. The sequence was 4-Cl > 3-Cl > 2-Cl. The result of different bases on the selectivity showed that NaOH had the lowest effect on the molar ration of the title compounds (Table S2). The different molar ratio of reagents was also an important factor to the reactive selectivity. The excessive acyl chloride made the diacylhydrazide as the byproduct, and the optimized molar ratio of acyl chloride and hydrazine was 1:4 in this case (Table S3).

**Figure 5:**
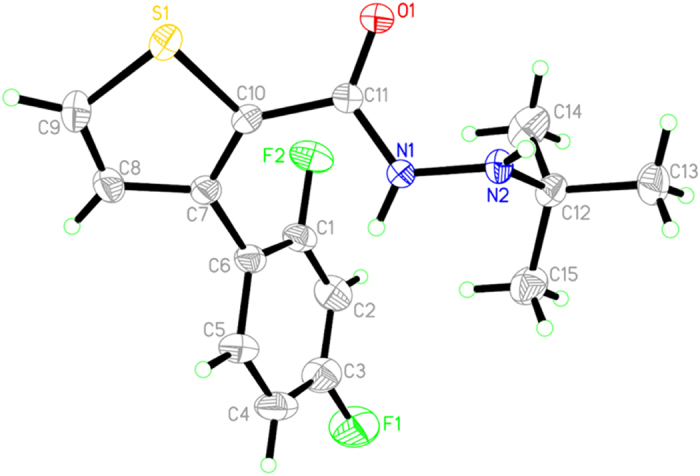
Molecular structure of **I7**. Displacement ellipsoids are drawn at the 30% probability level.

The crystal data are presented in Table S4 and Fig. 5, which give perspective view of compound **I7.** Some important bond lengths, angels, and torsion angles of compound **I7** are given in Table S5. It could be seen from the X-ray single crystal analysis of **I7** that the distance of C12-N2 (1.502(3) Å) and N1-N2 (1.426(3) Å) are equal to the van der Waals’ radii for C–N single bond (1.49 Å) and N-N single bond (1.45 Å) respectively. The single bond C11-N1 (1.349(3) Å) is typically the van der Waals’ radii for C–N double bond (1.35 Å), and the single bonds C6-C7 and C10-C11 (1.487(4) and 1.499(3) Å) are shorter than the standard C–C single bond (1.54 Å), but longer than C–C double bond (1.34 Å). All of these clearly indicated that the *p* orbital of N1 atoms conjugated with the π molecular orbital and formed the delocalized π-bonds with the conjoint thiophene and benzene ring. But the *p* orbit of N1 seemed not to be conjugated with the π molecular orbital of the C11–O1 double bonds, which was explained by the bond length of C11-O1 (1.227(3) Å) that followed in the normal range of C–O double bond (1.19–1.23 Å).

In the crystal of the structure, C (1), C (2), C (3), C (4), C( 5) and C (6) formed a plane with the mean deviation of 0.0074 Å, defined as plane I; C (7), C (8), C (9), C( 10) and S (1) formed a plane with the mean deviation of 0.0035 Å, defined as plane II; O (1), C (11), N (1), and N (2) were nearly coplanar with the mean deviation of 0.0162 Å, defined as plane III; C (12), C (13), C (14) and C (15) (the *tert*-butyl group) were not coplanar, which were defined factitiously as plane IV with the mean deviation of 0.3825 Å (Fig. 5 and Table S6). Plane II, plane III and plane IV make a dihedral angle with plane I of 58.9°, 69.4° and 83.0°. Plane III and plane IV make a dihedral angle with plane II of 40.1° and 113.6°. And the dihedral angle between plane III and plane IV is 149.3°. The related data are summarized in Table S6.

### Bioassay

#### Insecticidal Activity

**Figure 6:**
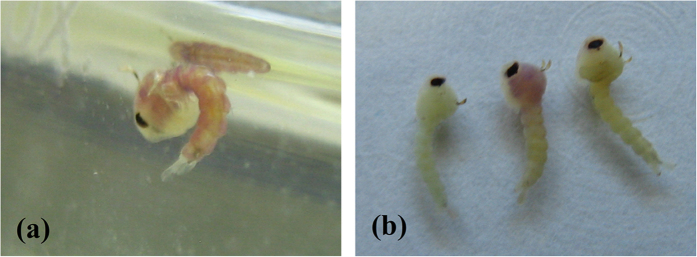
Comparison of the appearance of a control untreated *Culex pipiens pallens* pupa (**a**) with that of larval-pupal intermediates (**b**) treated with **I7**.

**Figure 7:**
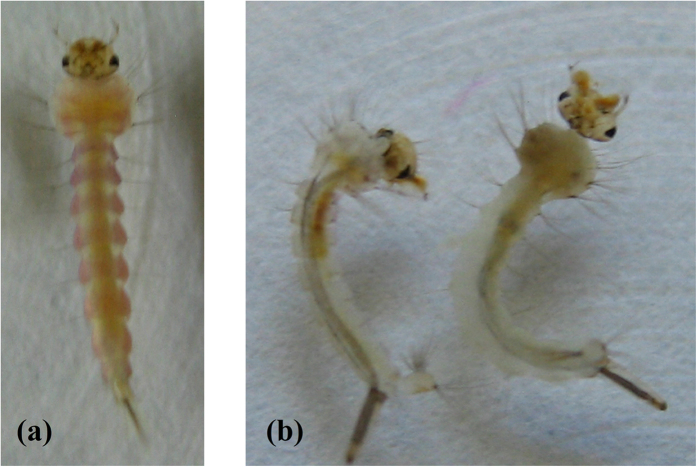
Comparison of the appearance of a control *Culex pipiens pallens* larva (**a**) with that of larvae (**b**) treated with **I7**.

The insecticidal results of the title compounds against *Plutella xylostella, Mythimna separata*, and *Culex pipiens pallens* were listed in Table 1. Mostly the insecticidal activity of compounds **I** was better than that of compounds **II**. The results indicate that the title compounds have significant promise for control of mosquitoes. For example, the LC_50_ of compounds **I1** and **I7** against *Culex pipiens pallens* were 2.47 ± 0.14 μg mL^−1^ and 2.33 ± 0.12 μg mL^−1^, which had the comparable activity with RH-5849 (2.52 ± 0.11 μg mL^−1^). The developmental effects on the mosquito larvae showed that the compounds can induce the formation of nonviable larval-pupal intermediates (Fig. 6). Examination of the mosquito larvae treated by the title compounds revealed the presence of an ecdysial space between the epidermis and cuticle (Fig. 7). In some cases, a complete new cuticle appeared to be produced in response to treatment with the compounds, but the larvae failed to shed the head capsule and ecdyse and died trapped within the exuvium.

Some title compounds also exhibited significant insecticidal activity against *Mythimna separate* and *Plutella xylostella*. For example, the LC_50_ of compound **I7** against *Mythimna separate* was 26.23 ± 0.89 μg mL^−1^, which was similar with RH-5849 (29.15 ± 1.08 μg mL^−1^). The LC_50_ of compound **I7** against *Plutella xylostella* was 80.24 ± 2.98 μg mL^−1^, which had the comparable activity with RH-5849 (79.32 ± 2.63 μg mL^−1^). Compounds **I1** and **I3** also showed good activity (LC_50_ were 34.39 ± 1.85 and 43.29 ± 2.31 μg mL^−1^) against *Mythimna separate*.

#### Growth inhibition activities

Considering the regulation of ecdysteroid analogs on development, growth, and metamorphosis, the effects of the compounds **I1** and **I7** after a 72 h treatment on the weight gain of larvae and inhibitory rates (Table 2), the duration of the pupal and larval stages, and also the eclosion rate of the treated *Culex pipiens pallens* were evaluated at 10 μg mL^−1^ (Table 3). The weight gain of larvae was inhibited by compounds **I1** and **I7**, which gave inhibitory rates of 37.8 ± 2.9% and 39.1 ± 2.7%. The effects of compounds **I1** and **I7** on the duration of the pupal and larval stages were not obvious, but the rates of eclosion were only 58.2 ± 3.1% and 60.1 ± 2.8% after treatment by compounds **I1** and **I7**. The results showed that compounds **I1** and **I7** possessed potent inhibitory activity on the growth and development of *Culex pipiens pallens*.

#### Reporter gene assay for the title compounds

**Figure 8:**
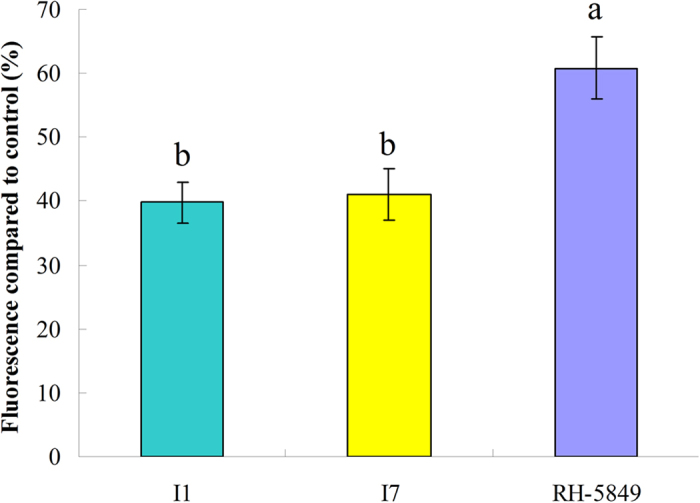
Reporter gene assay to evaluate molting hormone activity of compounds **I1** and **I7**. Compounds were assayed at 10 μg mL^−1^. The values were the means of three replicates and the error bars present the SE.

To confirm the bioactivity of the title compounds as ecdysteroid agonists, reporter gene assay^57^ was applied on compounds **I1** and **I7**. The induced fluorescence intensity was evaluated (Fig. 8). The reporter plasmid had an ERE (ecdysone response element) derived from the *Drosophila melanogaster* hsp27 gene and a basal actin promoter derived from *Bombyx mori*, which was followed by a gfp gene and a termination signal. In this study, the plasmid was transfected into Sf9 cells in which endogenous EcR and USP were detected. This cell-based reporter gene system could evaluate whether or not the tested compounds act on the receptor of EcR. After correction for auto-fluorescence, the fluorescence intensities of compounds **I1** and **I7** were higher than that of the negative control at 10 μg mL^−1^, which was no significant difference between each other. Although their fluorescence intensities were lower than that of RH-5849. This result suggested that the title compounds induced transcription of the reporter gene and acted on EcR as agonists.

#### Molecular modeling and docking study

**Figure 9:**
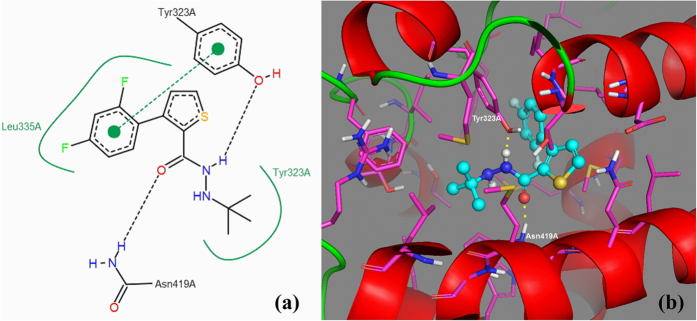
The 2D (**a**) and 3D (**b**) interaction modes of compound **I7** within the binding site of SlEcR LBD.

At least 4 residues in the LBD were involved in the interactions between the ligands and the receptor (Fig. 9). Compound **I7** could form hydrogen bonds with Tyr323 and Asn419 in the LBD interacting with the imine and carbonyl groups of **I7**. The hydrophobic *t*-butyl and 3-phenyl-2-thiophene group of **I7** were surrounded by the hydrophobic residues Tyr323 and Leu335, respectively. Meanwhile, the benzene ring of 3-phenyl-2-thiophene formed the π-π interaction with the residue Tyr323. The binding modes of **I7** within the LBD provide detailed structural insights into the interaction between the title compounds and the receptor. The formation of hydrogen bonds in the ligand-receptor complex, the hydrophobic and π-π interaction between the compounds and the ecdysone receptor EcR, which played key roles in promoting the binding affinity of the compound to regulate and disrupt the growth or sterility of the insects. That could lead to and promote the death of the insects.

## Conclusions

In summary, a pair of chemical isomeric structures of novel *N*-*tert*-butyl hydrazide containing 3-phenyl-2-thiophene moiety (**I** and **II)** were designed and synthesized. Their insecticidal tests indicated that most of the title compounds showed insecticidal activity against *Plutella xylostella, Mythimna separata*, and *Culex pipiens pallens*. Mostly the insecticidal activity of compounds **I** was better than that of compounds **II**. Some compounds **I** had excellent larvicidal activity against *Culex pipiens*. The title compounds functioned as ecdysteroid agonists, causing abnormal metamorphosis in insects and inducing transcription of the reporter gene. The observed biological effects on the mosquito larvae showed that the active compounds can induce the formation of nonviable larval-pupal intermediates. In some cases, mosquito larvae initiated molting and apolysis but failed to complete the molt and died trapped within the exuvium. The bioassay results showed that compounds **I** had great promise as a novel lead compound as insect growth regulators for further development.

## Additional Information

**How to cite this article**: Song, G.-P. *et al*. Synthesis and Larvicidal Activity of Novel Thenoylhydrazide Derivatives. *Sci. Rep.* **6**, 22977; doi: 10.1038/srep22977 (2016).

## Supplementary Material

Supplementary Information

## Figures and Tables

**Table 1:** Larvicidal Activity of Title Compounds#.

Compd.	R	Plutella xylostella		Mythimna separata		Culex pipiens pallens	
		Larvicidal activity (%) at 200 μg mL ^−1^	LC_50_ μg mL^−1^	Larvicidal activity (%) at 200 μg mL^−1^	LC_50_ μg mL^−1^	Larvicidal activity (%) at 10 μg mL^−1^	LC_50_ μg mL^−1^
I1	2-Cl	100 ± 3 a	91.23 ± 3.04 a	100 ± 4 a	34.39 ± 1.85 b	100 ± 3 a	2.47 ± 0.14 f
II1		80 ± 2 e		90 ± 3 b		100 ± 2 a	5.36 ± 0.35 b
I2	3-Cl	85 ± 2 d		80 ± 2 d		80 ± 3 c	
II2		80 ± 3 e		70 ± 2 f		60 ± 2 g	
I3	4-Cl	95 ± 2 b		100 ± 3 a	43.29 ± 2.31 a	100 ± 4 a	3.75 ± 0.21 e
II3		85 ± 2 d		85 ± 3 c		100 ± 3 a	7.25 ± 0.36 a
I4	2-F	90 ± 3 c		80 ± 2 d		100 ± 3 a	4.23 ± 0.15 d
II4		80 ± 2 e		75 ± 2 e		80 ± 3 c	
I5	3-F	75 ± 3 f		60 ± 2 h		70 ± 2 e	
II5		75 ± 2 f		65 ± 2 g		60 ± 2 g	
I6	4-F	90 ± 3 c		90 ± 3 b		100 ± 3 a	4.89 ± 0.25 c
II6		75 ± 2 f		80 ± 2 d		85 ± 3 b	
I7	2,4-di-F	100 ± 4 a	80.24 ± 2.98 b	100 ± 3 a	26.23 ± 0.89 c	100 ± 3 a	2.33 ± 0.12 f
II7		95 ± 2 b		90 ± 2 b		100 ± 2 a	4.32 ± 0.23 d
I8	2,6-di-F	80 ± 2 e		90 ± 3 b		70 ± 3 e	
II8		80 ± 3 e		80 ± 2 d		60 ± 2 g	
I9	2-NO_2_	75 ± 2 f		50 ± 2 j		50 ± 2 i	
II9		65 ± 2 h		60 ± 2 h		40 ± 2 k	
I10	3-NO_2_	50 ± 2 k		70 ± 3 f		60 ± 2 g	
II10		70 ± 2 g		65 ± 2 g		55 ± 2 h	
I11	4-NO_2_	65 ± 2 h		60 ± 2 h		75 ± 3 d	
II11		70 ± 3 g		75 ± 3 e		60 ± 2 g	
I12	H	60 ± 2 i		70 ± 2 f		65 ± 3 f	
II12		55 ± 2 g		65 ± 2 g		45 ± 1 j	
I13	4-CH_3_	60 ± 2 i		50 ± 2 j		50 ± 2 i	
II13		75 ± 3 j		45 ± 2 k		75 ± 3 d	
I14	4-OCH_3_	75 ± 3 i		65 ± 3 g		40 ± 2 k	
II14		65 ± 2 h		55 ± 2 i		45 ± 2 g	
I15	4-Br	55 ± 2 j		45 ± 2 k		30 ± 1 l	
II15		45 ± 2 l		50 ± 2 g		25 ± 1 m	
RH-5849		100 ± 3 a	79.32 ± 2.63 b	100 ± 3 a	29.15 ± 1.08 c	100 ± 3 a	2.52 ± 0.11 f

^#^Data derived from the mean of three independent assays (means ± SE). The same letters in the same column indicate no significant difference of the means at p < 0.05 by Duncan’s multiple-range test.

**Table 2:** The Effect of I1 and I7 on the Growth of *Culex pipiens pallens* #.

Compd.	Weight (mg)		Weigh gain (mg)	Inhibition (%)
	0 h	72 h		
**I1**†	11.7 ± 0.2 b	19.9 ± 0.4 b	8.3 ± 0.3 b	37.8 ± 2.9 a
**I7**†	12.2 ± 0.3 a	20.5 ± 0.6 b	8.6 ± 0.2 b	39.1 ± 2.7 a
Black control	11.5 ± 0.2 b	25.8 ± 0.5 a	13.8 ± 0.3 a	

^#^Data derived from the mean of three independent assays (means ± SE). The same letters in the same column indicate no significant difference of the means at *p* < 0.05 by Duncan’s multiple-range test.

^†^The concentration of **I1** and **I7** was 10 μg mL^−1^.

**Table 3:** The Effect of I1 and I7 on the Growth and Development of *Culex pipiens pallens* ^a^.

Compd.	Duration of lavae# (h)	Duration of pupae (h)	Rate of eclosion (%)
**I1**†	77.1 ± 4.1 a	30.1 ± 2.1 a	58.2 ± 3.1 b
**I7**†	75.2 ± 3.2 a	31.5 ± 2.6 a	60.1 ± 2.8 b
CK	74.5 ± 2.9 a	30.8 ± 2.5 a	96.7 ± 3.5 a

^#^Data derived from the mean of three independent assays (means ± SE). The same letters in the same column indicate no significant difference of the means at *p* < 0.05 by Duncan’s multiple-range test.

^†^The concentration of **I1** and **I7** was 10 μg mL^−1^. CK is the black control not containing the compounds.
