# Supplementary material for: Synthesis and Larvicidal Activity of Novel Thenoylhydrazide Derivatives
Source: Sci Rep. 2016 Mar 10;6:22977. doi: 10.1038/srep22977 (PMC4785375; doi:10.1038/srep22977)
Supplement: Supplementary Information [file srep22977-s1.doc]

**Synthesis and Larvicidal Activity of Novel Thenoylhydrazide Derivatives**

Gao-Peng Song^1,2,#^, De-Kun Hu^1,#^, Hao Tian^1,#^, Ya-Sheng Li^1^, Yun-Shen Cao^1^, Hong-Wei Jin^3, *^, Zi-Ning Cui ^1, *^

^1^ Guangdong Province Key Laboratory of Microbial Signals and Disease Control, South China Agricultural University, Guangzhou, 510642, China

^2^ College of Materials and Energy, South China Agricultural University, Guangzhou 510642, China

^3^ State Key Laboratory of Natural and Biomimetic Drugs, School of Pharmaceutical Sciences, Peking University, Beijing 100191, China

**Pages S3: Figure S1.** ^1^H NMR spectrum of compound **I3**;

**Figure S2.** ^1^H NMR spectrum of compound **II3**

**Pages S4: Figure S3.** ^1^H NMR spectrum of compound **I4;**

**Figure S4.** ^1^H NMR spectrum of compound **II4**

**Pages S5: Figure S5.** ^1^H NMR spectrum of compound **I6**;

**Figure S6.** ^1^H NMR spectrum of compound **II6**

**Pages S6: Figure S7.** ^1^H NMR spectrum of compound **I7**;

**Figure S8.** ^1^H NMR spectrum of compound **II7**

**Pages S7: Figure S9.** ^1^H NMR spectrum of compound **I8**;

**Figure S10.** ^1^H NMR spectrum of compound **II8**

**Pages S8: Table S1.** Effect of Different Substituted Groups on the Molar Ratio of Title Compounds **I** and **II**.

**Pages S9: Table S2.** Effect of Different Bases on the Molar Ratio of T itle Compounds **I** and **II***.*

**Pages S10: Table S3.** Effect of Different Molar Ratio of Reagents on the

Molar Ratio of Title Compounds **I** and **II**.

**Pages S11: Table S4.** Crystal and Experimental Data of Compound **I7**.

**Pages S12: Table S5.** Selected Bond Lengths, Angels, and Torsion Angles of Compound **I7**.

**Pages S13: Table S6.** Dihedral Angles and Mean Deviation of the Planes

in Compound **I7**.

To whom Correspondence should be addressed. Telephone/Fax: +86-20-85288229. E-mail: [ziningcui@scau.edu.cn](mailto:ziningcui@scau.edu.cn) (Z. C.), [jinhw@bjmu.edu.cn](mailto:jinhw@bjmu.edu.cn) (H. J.)

^#^These authors contributed equally to this paper.

**Figure S1.** ^1^H NMR spectrum of compound **I3**

**Figure S2.** ^1^H NMR spectrum of compound **II3**

**Figure S3.** ^1^H NMR spectrum of compound **I4**

**Figure S4.** ^1^H NMR spectrum of compound **II4**

**Figure S5.** ^1^H NMR spectrum of compound **I6**

**Figure S6.** ^1^H NMR spectrum of compound **II6**

**Figure S7.** ^1^H NMR spectrum of compound **I7**

**Figure S8.** ^1^H NMR spectrum of compound **II7**

**Figure S9.** ^1^H NMR spectrum of compound **I8**

**Figure S10.** ^1^H NMR spectrum of compound **II8**

**Table S1.** Effect of Different Substituted Groups on the Molar Ratio of Title Compounds **I** and **II**

| Entry | R | Base | T/^o^C | t/h | **Reagent**  **n _acyl chloride_/n _hydrazine_** | Product  n**_I_**/n**_II_** |
| --- | --- | --- | --- | --- | --- | --- |
| 1 | H | NaOH | 25 | 5 | 1/4 | 3.71/1 |
| 2 | 2-Cl | NaOH | 25 | 5 | 1/4 | 2.25/1 |
| 3 | 3-Cl | NaOH | 25 | 5 | 1/4 | 3.00/1 |
| 4 | 4-Cl | NaOH | 25 | 5 | 1/4 | 3.56/1 |
| 5 | 4-F | NaOH | 25 | 5 | 1/4 | 2.68/1 |
| 6 | 4-NO_2_ | NaOH | 25 | 5 | 1/4 | 3.27/1 |
| 7 | 4-CH_3_ | NaOH | 25 | 5 | 1/4 | 3.96/1 |
| 8 | 4-OCH_3_ | NaOH | 25 | 5 | 1/4 | 4.22/1 |

**Table S2. Effect of Different Bases on the Molar Ratio of Title Compounds I and II**

| Entry | R | Base | T/^o^C | t/h | Reagent  **n _acyl chloride_/n _hydrazine_** | Product  n**_I_**/n**_II_** |
| --- | --- | --- | --- | --- | --- | --- |
| 1 | 4-Cl | MeONa | 25 | 5 | 1/4 | 5.34/1 |
| 2 | 4-Cl | N(Et)_3_ | 25 | 5 | 1/4 | 4.21/1 |
| 3 | 4-Cl | K_2_CO_3_ | 25 | 5 | 1/4 | 4.56/1 |
| 4 | 4-Cl | NaOH | 25 | 5 | 1/4 | 3.56/1 |

**Table S3.** Effect of Different Molar Ratio of Reagents on the Molar Ratio of Title Compounds **I** and **II**

| Entry | R | Base | T/^o^C | t/h | Reagent  **n _acyl chloride_/n _hydrazine_** | Product  n**_I_**/n**_II_** |
| --- | --- | --- | --- | --- | --- | --- |
| 1 | 4-Cl | NaOH | 25 | 5 | 2/1 | 0.59/1^a^ |
| 2 | 4-Cl | NaOH | 25 | 5 | 1/1 | 1.38/1^b^ |
| 3 | 4-Cl | NaOH | 25 | 5 | 1/2 | 2.33/1^b^ |
| 4 | 4-Cl | NaOH | 25 | 5 | 1/4 | 3.56/1 |
| 5 | 4-Cl | NaOH | 25 | 5 | 1/6 | 3.48/1 |

^a^ The diacylhydazide was the main product; ^b^ There was the byproduct of diacylhydazide

**Table S4.** Crystal and Experimental Data of Compound **I7**

| Empirical formula | C_15_H_16_F_2_N_2_OS |
| --- | --- |
| Formula weight | 310.36 |
| *T* | 294(2) K |
| Wavelength | 0.71073 Å |
| Crystal system | Triclinic |
| Space group | *P*1 |
| Unit cell dimensions | a = 7.472(4) Å, *α* = 106.564(8)°  b = 10.557(5) Å, *β* = 102.836(7)°  c = 11.814(6) Å, *γ* = 108.513(8)° |
| Volume | 795.3(7) Å^3^ |
| *Z* | 2 |
| *D_x_* | 1.296 mg m^-3^ |
| Absorption coefficient | 0.224 mm^-1^ |
| *F* (0 0 0) | 324 |
| Crystal dimensions | 0.24 x 0.20 x 0.12 mm |
| *θ* range for data collection | 1.92 to 25.02 |
| Completeness to *θ* = 25.02 | 98.9 % |
| Limiting indices | -8≤*h*≤8, -12≤*k*≤8,-11≤*l*≤14 |
| Reflection collected/unique | 4104 / 2771 [R(int) = 0.0188] |
| Absorption correction | Semi-empirical from equivalents |
| Max. and min. transmission | 0.9737 and 0.9483 |
| Data/restraints/parameters | 2771 / 0 / 193 |
| Goodness-of-fit on *F*^2^ | 1.055 |
| Final *R* indices [*I* ＞ 2*σ* (*I*)] | R_1_ = 0.0479, wR_2_ = 0.1258 |
| 2*θ*_max_ | 50.04° with Mo *K*_α_ |
| (Δ*ρ*)_max_ | 0.492 eÅ^-3^ |
| (Δ*ρ*)_min_ | -0.485 eÅ^-3^ |
| Program system | SHELXS-97, SHELXL-97 |
| Structure determination | Direct method |
| Refinement | Full-matrix least-squares on *F*^2^ |
| CCDC No. | 935115 |

**Table S5.** Selected Bond Lengths, Angels, and Torsion Angles of Compound **I7**

| Lengths | (Å) | Angles | (°) | Torsion angles | (°) |
| --- | --- | --- | --- | --- | --- |
| S(1)-C(10) | 1.723(3) | C(9)-S(1)-C(10) | 91.61(13) | C(7)-C(8)-C(9)-S(1) | 0.4(3) |
| O(1)-C(11) | 1.227(3) | C(11)-N(1)-N(2) | 121.72(18) | C(1)-C(6)-C(7)-C(10) | -58.9(3) |
| N(1)-C(11) | 1.349(3) | N(1)-N(2)-C(12) | 112.66(17) | C(1)-C(6)-C(7)-C(8) | 119.4(3) |
| N(1)-N(2) | 1.426(3) | C(8)-C(7)-C(6) | 122.3(2) | S(1)-C(10)-C(11)-O(1) | -38.4(3) |
| N(2)-C(12) | 1.502(3) | C(8)-C(9)-S(1) | 112.3(2) | S(1)-C(10)-C(11)-N(1) | 139.10(19) |
| F(1)-C(3) | 1.365(3) | C(7)-C(10)-C(11) | 130.9(2) | N(2)-N(1)-C(11)-O(1) | 5.8(4) |
| C(12)-C(14) | 1.529(4) | O(1)-C(11)-N(1) | 124.1(2) | N(2)-N(1)-C(11)-C(10) | -171.69(19) |
| C(10)-C(11) | 1.499(3) | O(1)-C(11)-C(10) | 121.8(2) | N(1)-N(2)-C(12)-C(13) | -177.46(19) |
| C(6)-C(7) | 1.487(4) | N(1)-C(11)-C(10) | 114.02(19) | N(1)-N(2)-C(12)-C(14) | 62.4(3) |
| C(7)-C(10) | 1.375(3) | N(2)-C(12)-C(13) | 105.4(2) | N(1)-N(2)-C(12)-C(15) | -60.6(2) |
| C(7)-C(8) | 1.431(4) | C(9)-C(8)-C(7) | 113.2(2) | C(11)-N(1)-N(2)-C(12) | -107.0(2) |

**Table S6.** Dihedral Angles and Mean Deviation of the Planes in Compound **I7**

| Dihedral angles (°) | |
| --- | --- |
| Plane I and plane II | 58.9 |
| Plane I and plane III | 69.4 |
| Plane I and plane IV | 83.0 |
| Plane II and plane III | 40.1 |
| Plane II and plane IV | 113.6 |
| Plane III and plane IV | 149.3 |
| The mean deviation of the plane (Å) | |
| Plane I (C1 to C6) | 0.0074 |
| Plane II C7 to C10, S1 | 0.0035 |
| Plane III O1, C11, N1, N2 | 0.0162 |
| Plane IV | 0.3825 |
